# Comparative Host Specificity of Human- and Pig- Associated *Staphylococcus aureus* Clonal Lineages

**DOI:** 10.1371/journal.pone.0049344

**Published:** 2012-11-14

**Authors:** Arshnee Moodley, Carmen Espinosa-Gongora, Søren S. Nielsen, Alex J. McCarthy, Jodi A. Lindsay, Luca Guardabassi

**Affiliations:** 1 Department of Veterinary Disease Biology, Faculty of Health and Medical Sciences, University of Copenhagen, Frederiksberg, Denmark; 2 Department of Large Animal Sciences, Faculty of Health and Medical Sciences, University of Copenhagen, Frederiksberg, Denmark; 3 Department of Clinical Sciences, St George's University of London, London, United Kingdom; University of Iowa, United States of America

## Abstract

Bacterial adhesion is a crucial step in colonization of the skin. In this study, we investigated the differential adherence to human and pig corneocytes of six *Staphylococcus aureus* strains belonging to three human-associated [ST8 (CC8), ST22 (CC22) and ST36(CC30)] and two pig-associated [ST398 (CC398) and ST433(CC30)] clonal lineages, and their colonization potential in the pig host was assessed by *in vivo* competition experiments. Corneocytes were collected from 11 humans and 21 pigs using D-squame® adhesive discs, and bacterial adherence to corneocytes was quantified by a standardized light microscopy assay. A previously described porcine colonization model was used to assess the potential of the six strains to colonize the pig host. Three pregnant, *S. aureus*-free sows were inoculated intravaginally shortly before farrowing with different strain mixes [mix 1) human and porcine ST398; mix 2) human ST36 and porcine ST433; and mix 3) human ST8, ST22, ST36 and porcine ST398] and the ability of individual strains to colonize the nasal cavity of newborn piglets was evaluated for 28 days after birth by strain-specific antibiotic selective culture. In the corneocyte assay, the pig-associated ST433 strain and the human-associated ST22 and ST36 strains showed significantly greater adhesion to porcine and human corneocytes, respectively (p<0.0001). In contrast, ST8 and ST398 did not display preferential host binding patterns. In the *in vivo* competition experiment, ST8 was a better colonizer compared to ST22, ST36, and ST433 prevailed over ST36 in colonizing the newborn piglets. These results are partly in agreement with previous genetic and epidemiological studies indicating the host specificity of ST22, ST36 and ST433 and the broad-host range of ST398. However, our *in vitro* and *in vivo* experiments revealed an unexpected ability of ST8 to adhere to porcine corneocytes and persist in the nasal cavity of pigs.

## Introduction

*Staphylococcus aureus* is able to colonize and cause infections in humans and a wide range of animals. In the 1970s, host-specific ecovars of *S. aureus* were described based on biotyping [1]–[3]. Subsequently, population genetic studies have identified genotypes that are associated with specific host species. For example, sequence type (ST)71, ST97, ST126, ST133 and ST151 among ruminants [4]–[8], ST5 in poultry [7], [9], and ST9, ST398 and ST433 in pigs [10]. In humans, the major lineages are ST1, ST5, ST8, ST22, ST30, ST36, ST45, ST57, ST80, ST228, ST239, ST247, and ST250 [11].

*S. aureus* adherence and colonization of the skin is a complex biological event requiring an optimal match between host and bacterium [12], [13]. Although *S. aureus* is generally regarded as a host-specific organism, there is increasing evidence that certain clonal lineages (e.g. ST22, ST254 and ST398) have extended-host spectrum and are able to adapt to both humans and animals [14], [15]. The current knowledge of the clonal host range of *S. aureus* is largely based on epidemiological and genetic studies. In the present study, we analyzed the *in vitro* adherence of six *S. aureus* strains belonging to three human-associated [ST8 (C8), ST22 (CC22) and ST36(CC30)] and two pig-associated [ST398 (CC398) and ST433(CC30)] clonal lineages to human and pig corneocytes, and their colonization potential in the pig host by *in vivo* competition experiments, in order to understand the host-specificity of major human and pig clonal lineages. We focused our attention on ST398 and ST433 as these are two of the most common lineages found in pigs [16]. Moreover, ST398 has also been isolated from human infections and is regarded as an important zoonotic agent [17]. ST8 (USA300), ST22 (EMRSA-15), and ST36 (EMRSA-16) were included in the study because they are typical human-associated lineages and had never been reported in pigs at the time of the study.

## Materials and Methods

### Strains

Six genetically distinct *S. aureus* strains isolated from the nasal cavity of healthy pigs and human infections were used. The six strains differed with respect to multi-locus sequence type (ST8, ST22, ST36, ST398 and ST433), *spa* type (t008, t011, t018, t032, t034 and t1333) and antimicrobial susceptibility profile (Table 1). The ST8, ST22 and ST36 strains were selected as representatives of human-associated lineages, whereas ST398 and ST433 were included as pig-associated lineages. As ST398 is frequently isolated from humans living in contact with livestock, a strain isolated from pig (PIL1) and a strain isolated from a human infection (PIL130) were included to study host adaptation within this lineage.

**Table 1:** Strains used in the experiment.

Strain name	Strain mix*	Country of origin	Host origin	*spa* type	ST type	CC	SCC*mec*	Resistance Phenotype						
PIL1	1 & 3	Belgium	Pig	t011	ST398	CC398	V	OX	TET	STREP				
PIL130	1	Belgium	Human	t034	ST398	CC398	MSSA				CAD			
PIL131	2 & 3	Denmark	Human	t018	ST36	CC30	II	OX			CAD			CHL
PIL135	2	Denmark	Pig	t1333	ST433	CC30	MSSA					CIP		
FPR3757	3	USA	Human	t008	ST8	CC8	IV	OX	TET			CIP	MUP	
PM84	3	United Kingdom	Human	t032	ST22	CC22	IV	OX	TET		CAD	CIP		

* Strain mixes used to inoculate the three sows in the *in vivo* colonization experiment.

OX, oxacillin; TET, tetracycline; CIP, ciprofloxacin; CAD, cadmium; STREPT, streptomycin; MUP, mupirocin; CHL, chloramphenicol; MSSA, methicillin susceptible *S. aureus*.

### Collection of corneocytes

Corneocytes were collected from 11 humans (age range: 28–38 years) and 21 pigs (age range: 7–8 weeks). All humans were negative for nasal carriage of *S. aureus* at the time of sampling and had not received antibiotics for at least one month before sampling. Eight pigs (38%) were *S. aureus*-negative at sampling, three pigs were naturally colonized with methicillin-resistant *S. aureus* (MRSA) ST398, and 10 pigs were from artificially colonized with different strain mixes in the *in vivo* colonization experiment described below. Human corneocytes were taken from the inner forearm and pig corneocytes were taken from the outer ear. Prior to sampling, surface debris was removed by applying three successive adhesive tape strips. Corneocytes were collected using 25-mm diameter adhesive discs (D-squame®, CuDerm Corporation, Dallas, USA) which were pressed onto the cleaned skin and then removed to obtain a confluent corneocyte layer. Twelve discs per individual were taken and the same investigator collected the samples in a standard manner in order to reduce sampling variability. Written informed consent was obtained and protocols approved by the Danish National Committee on Biomedical Research Ethics (H-KF-2007-0007).

### Adherence assay

From a 10 mL overnight culture in BHI incubated without shaking at 37°C, 2 mL was centrifuged (1500 g for 5 minutes) and washed three times in phosphate buffered saline (PBS) by vortexing and centrifugation (800 g for 3 minutes). The final suspension was adjusted to an optical density (OD)_600_ = 0.289 (∼3×10^8^ CFU/mL), and 500 µL of this OD-adjusted suspension was pipetted over the corneocyte layer and incubated at 37°C in a moist chamber for 45 min. After incubation, discs were washed with PBS, and stained for 30 sec with crystal violet, and washed again in PBS to remove excess stain, and left to air-dry. From each D-Squame® disc, ten digital images from ten confluent corneocytes were selected, with each image respresenting one area within one corneocyte. The number of adhering bacteria was counted manually at ×1000 magnification using the Axioplan II epifluorescence microscope (Zeiss, Oberkochen, Germany) and a Zeiss AxioCam digital camera. Negative control slides, one corneocyte disc per individual, were incubated with phosphate buffered saline only and included in every experiment [18], [19].

Counts of adherent bacteria for each microscope field were compared in a negative binomial regression model, which was done using the Genmod procedure in SAS version 9.2 (SAS Institute, Cary, NC, USA). This model was used to assess whether pig strains (PIL1, PIL135) were associated with adherence to pig corneocytes, human strains (PM84, FPR3757, PIL130, PIL131) were associated with adherence to human corneocytes, and vice versa. The mean bacterial count was estimated for each strain-corneocyte combination, and differences in log (mean adherence counts) were assessed by differences in least squares means for the interaction between host species (pig or human) and strain species (pig or human). To assess if *S. aureus* carrier status has an effect on the number of adherent bacteria to corneocytes, a similar approach was used where the differences in log (mean adherence counts) were compared among *S. aureus* carrier and non-carrier piglets. A *p*-value of <0.05 was considered a significant difference.

### *In vivo* pig host specificity using a vertical transmission colonization model

A colonization model based on sow vaginal inoculation [20] was used to test the ability of the six strains, representing five different lineages, to colonize piglets born from the inoculated sows. Three *S. aureus*-negative, 97-day pregnant sows were purchased from a specific-pathogen-free (SPF) farm in Denmark. After nine days acclimatization and shortly before farrowing, each sow was inoculated intravaginally with one of the three strain mixes (Table 1) as described by Moodley et al. [20]. No antibiotics were used to aid colonization. The experiment was carried out in class II isolation facilities at the Faculty of Health and Medical Sciences, University of Copenhagen, and all the procedures used were performed in compliance with the Animals Scientific Act, and approved by the Danish Animal Experimentation Inspectorate (License no. 2006/561-1141).

Nasal swabs were collected from both nostrils of each piglet usinga single swab on Days 0 (farrowing), 7, 14, 21 and 28 (end of the experiment). To determine the stability of colonization and assess the effects of environmental contamination, piglets were removed after the last sampling (Day 28) and placed in two new, sterile rooms, and were sampled once prior to termination (Day 35). Swabs were enriched in 5 mL Mueller Hinton broth (MHB) containing 6.5% NaCl and incubated overnight at 37°C. Thereafter, 10 µL of the enrichment broth was streaked onto Oxacillin Resistance Screening Agar Base (ORSAB, Oxoid) plates containing combinations of oxacillin (4 µg/mL), streptomycin (1000 µg/mL), chloramphenicol (16 µg/mL), ciprofloxacin (2 µg/mL), cadmium (32 µg/mL) or mupirocin (16 µg/mL) to enable detection of the different strains based on their susceptibility profiles (Table 1). After overnight incubation at 37°C, one denim blue colony per plate was sub-cultured on 5% blood agar and strains exhibiting *S. aureus* colony morphology were confirmed to be the same genotype as the inoculated strain using a restriction modification PCR for strains isolated from the mixed clonal complex (CC) inoculated group [21], [22] or strain-specific PCRs, which were developed to differentiate between the CC398 and CC30 isolates, respectively. To differentiate between the CC398 isolates (PIL1 and PIL30), primers (phi3F: 5′-TGAAAACACGTTGTTACGATGG-3′ and phi3R: 5′-ATCCGCCTTCTTTGAAAATGTA-3′) were designed to target the integrase (*int*) gene of the prophage φ3, which is present only in the human CC398 isolate (PIL30) [23]. To differentiate between the two CC30 isolates (PIL131 and PIL135), primers (sraPF: 5′GCAAATCAGCATCAACTGCA3′ and sraPR: 5′TTGAGTCACCTGTATCTGGCA3′) were designed to detect the presence of a truncated *sraP*, which is only found in the human CC30 isolate (PIL131). Colonization was defined as five consecutive positive cultures over four weeks.

## Results

### *In vitro* corneocyte adherence assay

**Figure 1:**
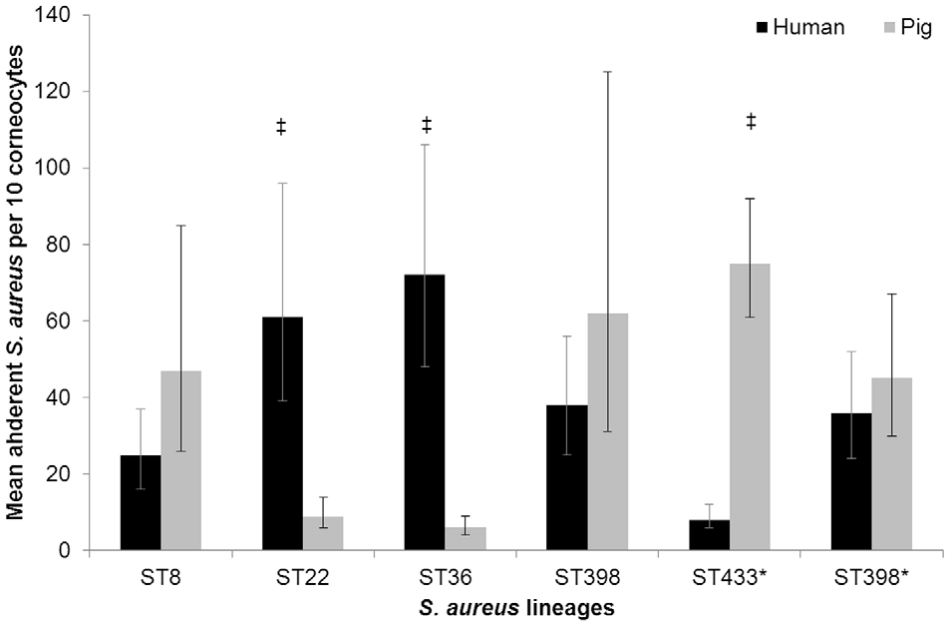
Mean adherence of the different *S. aureus* isolates to human and pig corneocytes. Bars represent the back transformed mean adherence and the error bars indicate the 95% confidence intervals. *isolates of pig origin. ‡significantly better adherence to porcine and human corneocytes, respectively.

**Figure 2:**
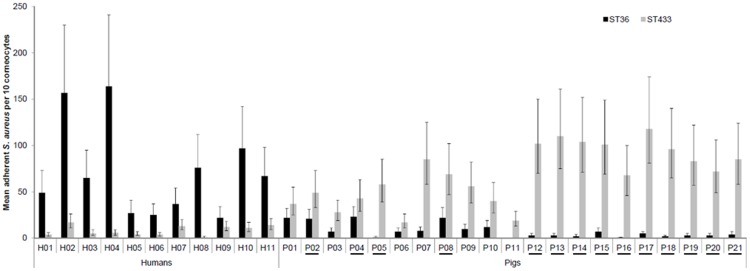
Mean adherence of human ST36 (CC30) and pig ST433 (CC30) to human and pig corneocytes. Bars represent the mean, and the error bars indicate the 95% confidence intervals. H1–H10; corneocytes from healthy humans. P1–P21; corneocytes from healthy pigs. Underlined pig numbers indicate positive *S. aureus* status.

The mean adherence of the different *S. aureus* isolates to human and pig corneocytes are shown in Figure 1. Overall, porcine isolates showed significantly greater adhesion to porcine corneocytes than to human corneocytes (*p*<0.0001), whereas, isolates adhered better to human corneocytes (*p*<0.0001). An example of this host-specific preferential binding is illustrated in Figure 2. The pig-associated ST433 strain adhered better to pig corneocytes than to human corneocytes (*p*<0.0001), whereas, the human-associated ST36 adhered better to human than to pig corneocytes (*p*<0.0001). Similarly, a significant difference was observed for ST22, which adhered better to human than to pig corneocytes (*p*<0.0001). ST8 and ST398 strains adhered slightly better to pig corneocytes than human corneocytes but this was not statistically significant. No significant difference was observed between the binding patterns of the porcine and human ST398 strains. Amongst pigs, positive *S. aureus* carriers had significantly more adherent bacteria (mean count of all six isolates = 40 bacteria) compared to non-carriers (mean count of all six isolates = 31 bacteria). No bacteria were observed in the negative control slides as a consequence of surface debris removal prior to collection of corneocytes.

### *In vivo* colonization

**Figure 3:**
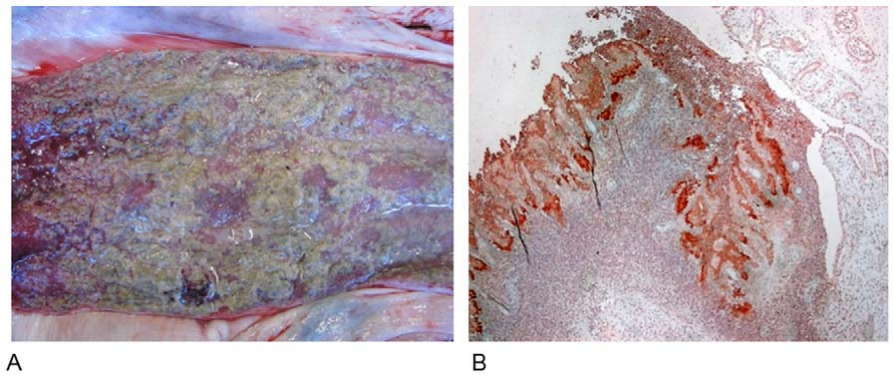
Necropsy and stained tissue section of the uterus in the sow inoculated with CC398. A) Body of uterus. Sub-acute necrotizing and purulent endometritis. B) Uterine horn. Acute necrotising and purulent endometritis. Bacterial colonies (red-brown colour of aminoethylcarbazol) identified as *S. aureus* by immunohistochemistry.

In the first experiment, the sow inoculated with the human and pig ST398 strains (mix 1; PIL1 and PIL130) developed acute necrotising and purulent endometritis, resulting in abortion of the piglets. Both the human and pig strain were cultured from necropsy samples from the reproductive organs and immunohistochemistry confirmed presence of *S. aureus* in the tissue sections (Figure 3A and B). Results for the remaining pig experiments are shown in Table 2. The eight piglets born from the sow inoculated with the two strains belonging to CC30 (mix 2) were colonized with the pig-associated lineage (ST433) for the entire duration of the experiment, whereas the human-associated lineage (ST36) was only detected at farrowing. In the third experiment, partum of the sow inoculated with ST8, ST22 and ST36 of human origin and ST398 of pig origin (mix 3) resulted in 11 piglets. Surprisingly, ST8 was the only strain consistently isolated from the nasal cavity of all piglets. ST22 was only present in the first two weeks after farrowing. The ST398 strain of porcine origin was only detected in one piglet on two separate sampling days. ST36 was not detected in any of the piglets. After Day 28, eight piglets positive for ST433 and ST8 were placed in two separate sterile rooms, respectively to assess stability of colonization. Only piglets positive for ST433 remained positive after one week, whereas piglets harbouring ST8 were negative on Day 35.

**Table 2:** Number of positive piglets (percentage) for the various *S. aureus* strains used in the *in vivo* colonization experiment.

	Day 0	Day 7	Day 14	Day 21	Day 28	Day 35
Piglets from Sow 2 inoculated with CC30 (n = 8)						
ST36	8 (100%)	0 (0%)	0 (0%)	0 (0%)	0 (0%)	0 (0%)
ST433	8 (100%)	8 (100%)	8 (100%)	8 (100%)	8 (100%)	8 (100%)
Piglets from Sow 3 inoculated with mixed CCs (n = 11)						
ST8	11 (100%)	11 (100%)	11 (100%)	11 (100%)	11 (100%)	0 (0%)
ST22	11 (100%)	9 (82%)	0 (0%)	0 (0%)	0 (0%)	0 (0%)
ST36	0 (0%)	0 (0%)	0 (0%)	0 (0%)	0 (0%)	0 (0%)
ST398	0 (0%)	1 (9%)	0 (0%)	1 (9%)	0 (0%)	0(0%)

## Discussion

Adherence is the first step for bacterial colonization of a host skin. Corneocytes are the outermost layer of the epidermis and are part of the vital barrier function of the skin. In this study, we have used an experimental approach to quantify adherence to porcine and human corneocytes by five *S. aureus* clonal lineages. The results were generally in agreement with apparent host range of the selected lineages.

Due to the abortion of all piglets inoculated with ST398 (experiment 1), host specificity could not be investigated. However, in the adherence assay, both the human and the porcine ST398 strains did not have an overall preferential binding to either humans or pigs corneocytes, suggesting a broader host range. The two ST398 strains used in this study were included in a microarray-based comparative genomic study of human and pig ST398 isolates from Europe to identify unique genes or gene variants associated with host specificity or adaptation [23]. It was shown by microarray analysis [23] and subsequently whole genome sequencing [24] that there was no unique gene that was associated with either human or pig host specificity. However, many human ST398, but few pig ST398 isolates harboured the human immune evasion cluster (IEC) genes, which are carried on the prophage φ3 [23], [24]. Uhlemann et al. [25] recently compared the genomes and adherence to skin keratinocytes of pig MRSA ST398 and MSSA ST398 from humans with no pig contact. The genomes differed in MGE content and variations in surface adhesion genes. Compared to the pig MRSA ST398, the human MSSA ST398 bound significantly better to human keratinocytes. No significant difference was observed between the human and pig ST398 and adherence to pig keratinocytes [25]. ST398 is a common member of the commensal flora in pigs [15] but rarely causes clinical infections in these animals. The presence of both ST398 strains in the reproductive tract lesions of the sow provides experimental evidence that this lineage may cause genital tract infections leading to abortion of the piglets. However, it should be noted that the high bacterial inoculum used in the experiment may not reflect natural field conditions. While *S. aureus* is generally not regarded as a pig pathogen, this species is frequently isolated from lesions observed during post-mortem inspection, especially abscess in the lung [26] and the udder [27].

Within CC30, the preferential binding of ST433 to porcine corneocytes and of ST36 to human corneocytes is perfectly in line with the natural hosts of these *S. aureus* lineages, which are pig and human, respectively. The host specificity of these two genetically-related lineages was confirmed by the *in vivo* colonization experiment, where the ST433 was better at colonizing piglets compared to the ST36, and ST433 colonization was stable after piglets were placed in a sterile environment ruling out environmental recontamination. These results indicate that these two STs, despite belonging to the same CC (CC30), have evolved separately and adapted to the different host species. ST433 is a double locus variant of ST36 and based on microarray analysis, the two strains harbour different mobile genetic elements (MGEs) that may contribute to host specificity and host tropism (data not shown).

Similar to ST36, the ST22 strain exhibited reduced binding to pig corneocytes compared to human corneocytes suggesting a preference of this clonal lineage for adhering to human corneocytes. This observation is supported by inability of the strain to colonize piglets in the comparative *in vivo* host specificity experiment as well as by the current epidemiological information supporting ST22 colonisation of humans and not pigs. Both the ST36 and ST22 strains used are examples of EMRSA-15 and EMRSA-16 which are human epidemic hospital-associated MRSA (HA-MRSA) [28] and to our best knowledge, have never been reported in pigs. ST22 asymptomatic carriage and infections have been reported in companion animals in the UK [29], Ireland [30] and Germany [31], where this clone is prevalent in the human population. The occurrence of EMRSA-15 in companion animals is hypothesized to be a consequence of transmission from humans [4], [29], [32].

Similarly to ST398, ST8 (USA 300) did not demonstrate preferential binding to corneocytes from either humans or pigs. Surprisingly, this lineage outcompeted the other three strains present in the inoculum mix, including the pig ST398 strain, in the colonization experiment. No antagonism between these strains (ST8, ST22, ST36 and ST398) could be detected *in vitro* by a) measuring inhibition zone diameters after spotting supernatant derived from an centrifuged overnight culture of one strain onto a spread plate of the other strain, and b) growing two strains together in liquid medium and measuring the CFU/mL of each strain at 1 h intervals for 6 hrs (data not shown). However, *in vivo* colonization could be influenced by bacterial interference with other staphylococcal species present in the normal flora of the pigs, as suggested by a recent study of MRSA ST398 colonization in gnotobiotic piglets [33]. It is also a possibility that the undetected strains could have been present at low numbers below the detection limit of the method and/or have a slower growth rate resulting in the strains being outgrown during enrichment by faster growing strains. ST8 is a well-known community-associated (CA)-MRSA and a common cause of skin and soft tissue infections amongst people with no known risk factors for MRSA infections. Asymptomatic carriage of ST8, also known as USA300, has recently been reported in pigs in the US [34], in one scavenging pig in Peru [35], and found in the internal organs from clinically healthy pigs in a nasal colonization experiment [36]. There has been one report of this CA-MRSA causing porcine soft tissue infection [37]. Despite our results showing that ST8 nasal colonization was not stable and piglets eliminated the strain when they were housed in a clean sterile environment, human ST8 displayed an unexpected ability to adhere to porcine corneocytes and persist in the nasal cavity of pigs. It cannot be excluded that ST8 was present on other body sites not sampled e.g. skin and perineum.Therefore, farm contamination with ST8 should be avoided to prevent possible transmission to pigs, which could become a potential reservoir of this human epidemic clone.

To elucidate the molecular mechanisms underlying *S. aureus* host specificity, comparative genome analyses of human and animal derived strains have been performed [4], [7], [38]–[40]. While there are differences in the MGE content, there is little difference between core variable genes i.e. genes encoding predicted surface proteins from animal and human *S. aureus* strains, suggesting that *S. aureus* proteins are able to interact with host proteins from different host species. This may explain why some lineages are able to colonize and cause infections in both animals and humans [23], [40]. We observed a clear effect of strain type and individual host, meaning that corneocytes from each individual bind different strains uniquely. This would be expected since each strain is likely to produce different adhesins and each individual would have different ligands on the cell surfaces. Strain variation and differential adherence to human fibronectin has previously been shown for *S. aureus* [41]. Furthermore, we observed that strains adhere significantly better to corneocytes from pig *S. aureus* carriers compared to non- *S. aureus* carriers. Nouwen et al. [12] demonstrated that human non-carriers could not be colonized when experimentally challenged with *S. aureus* indicating that host genetics co-determine the carrier state.

There are three major limitations in this study. In the colonization experiment, only a single sow was used for each experiment, and in the corneocyte adherence assay the results are based on the mean adherence of 10 corneocytes in one experiment. The corneocyte adherence assay has previously been shown to be reproducible [19], [42] and has been used to investigate host specificity of different staphylococcal species [18], [19], [43]. Lastly, only stationary phase bacterial cultures were used in the adherence assay. *S. aureus* cell wall adhesins and secreted virulence factors that play a role in adherence to mammalian cells are differentially expressed during the different growth phases [44]. Using only stationary phase cultures, we observed adherence as a result of those adhesins preferentially expressed in the stationary phase.

In conclusion, our results demonstrate that certain staphylococcal lineages are host specific, while others have a broader host range. This is supported by the current knowledge of the epidemiology and population structure of the five clonal lineages under study with the only exception of ST8 (US300). Surprisingly, ST8 did not have any preferential binding to human corneocytes and persisted for a long period in the nasal cavity of experimentally colonized piglets, suggesting a possible broad-host range of this CA-MRSA clone.
